# Two-Color Pixel Patterning for High-Resolution Organic Light-Emitting Displays Using Photolithography

**DOI:** 10.3390/mi11070650

**Published:** 2020-06-30

**Authors:** Yu Min Choi, Han Young Shin, Jongchan Son, Chunhee Park, Keun-Woo Park, Jin-Kyun Lee, Byung Jun Jung

**Affiliations:** 1 Department of Materials Science and Engineering, University of Seoul, Seoul 02504, Korea; ccwe1001@naver.com (Y.M.C.); tlsgksdud24@naver.com (H.Y.S.); 2 Department of Polymer Science and Engineering, Inha University, Incheon 22212, Korea; thswhdcks1@naver.com (J.S.); nhd8991@naver.com (C.P.); rmsdn0112@naver.com (K.-W.P.)

**Keywords:** organic light-emitting diode (OLED), photolithography, pixel patterning, high resolution, highly fluorinated materials

## Abstract

Nowadays, the display industry is endeavoring to develop technology to provide large-area organic light-emitting diode (OLED) display panels with 8K or higher resolution. Although the selective deposition of organic molecules through shadow masks has proven to be the method of choice for mobile panels, it may not be so when independently defined high-resolution pixels are to be manufactured on a large substrate. This technical challenge motivated us to adopt the well-established photolithographic protocol to the OLED pixel patterning. In this study, we demonstrate the two-color OLED pixels integrated on a single substrate using a negative-tone highly fluorinated photoresist (PR) and fluorous solvents. Preliminary experiments were performed to examine the probable damaging effects of the developing and stripping processes upon a hole-transporting layer (HTL). No significant deterioration in the efficiency of the develop-processed device was observed. Efficiency of the device after lift-off was up to 72% relative to that of the reference device with no significant change in operating voltage. The procedure was repeated to successfully obtain two-color pixel arrays. Furthermore, the patterning of 15 μm green pixels was accomplished. It is expected that photolithography can provide a useful tool for the production of high-resolution large OLED displays in the near future.

## 1. Introduction

The display industry has been evolving from liquid crystal displays (LCDs) towards organic light-emitting diode (OLED) technology that can implement paper-thin displays with excellent viewing angle and more vivid colors, and now OLED has successfully entered the commercialization stage. The basic structure of OLEDs was first published in 1987 by Ching, W. Tang and Steven Vanslyke, and these have since led to huge studies by many research groups [1,2,3,4]. At present, OLEDs are adopted not only for televisions, but also for mobile phones and other portable smart devices. In addition, innovative technologies, such as visualization of virtual reality (VR)/augmented reality (AR) and deformable displays that can be folded and stretched, are also being actively sought for with OLED technology [5,6,7].

Along with the challenges in extending the applications, the display industry is moving rapidly towards producing ultra-high-resolution (≥8K) large OLED displays [8,9,10]. To enhance the resolution of OLEDs, the number of pixels per unit area must be increased. Hence, the area occupied by red, green and blue (RGB) subpixels must be further decreased. The current commercial pixel patterning protocol for mobile OLEDs is selective deposition using fine metal masks (FMM method). A shadow mask composed of a thin metal plate and tiny holes perforated on it (FMM) is positioned below a substrate in a vacuum chamber, and electrically active layers, including a light-emitting layer (EML) of the desired color, is deposited only at the open regions of the FMM. This means that the holes on the mask must be extremely small and densely assembled if an ultra-high-resolution display is to be fabricated using the FMM method. Furthermore, it is required that the thickness of the metal plate must be thinner than the size of the pixels to avoid the shadow effect, and FMM must be sufficiently light to prevent it from sagging by its own weight. Production of such metal masks with these physical constraints is technically challenging and costly, thus limiting the production of an ultra-high-resolution OLED displays [11,12,13].

To overcome the limits of the FMM method, various RGB pixel patterning techniques such as ink-jet [14,15], organic vapor jet printing (OVJP) [16,17], laser-induced thermal imaging (LITI) [18,19], laser-induced pattern-wise sublimation (LIPS) [20], etc. have been studied. Recently, we have paid attention to photolithography as a potential scheme for vacuum-deposited OLED multicolor pixel patterning. Photolithography with its high-resolution capabilities and well-established registration method is the only reliable patterning tool for integrated circuits in the semiconductor industry. This technical benefit motivated several research groups to apply it to OLED pixel patterning. They used a bi-layer resist approach [21,22,23] or photo-crosslinkable electroluminescent polymers as EML [24]. In case of a bi-layer resist approach, the choice of shielding layer and photoresist is important. The complete removal of the shielding layer is essential and the proper processing time is desirable. Introducing a photo functional group to EML material is difficult because of a complex synthetic route.

Unlike general photosensitive materials that use organic solvents and aqueous developers, highly fluorinated photoresists (PR) can be processed with fluorous solvents which do not affect significantly the physical and electrical properties of the robust polymeric electronic materials or organic functional films made of pentacene [25,26]. In a previous report [27], the lift-off step of a resorcinarene-based PR film was carried out in a fluorous solvent mixture containing 1,1,1,3,3,3-hexamethyldisilazane (HMDS) or isopropyl alcohol (IPA). However, it is difficult to apply the protocol to the lift-off process of vacuum-deposited organic thin-films made of various OLED materials, in particular, EMLs consisting of small molecular hosts and Ir-based phosphorescent dyes. In this study, we performed OLED pixel patterning on a common hole-transport layer (HTL) using a newly developed, highly fluorinated PR [28] which is soluble in pure fluorous solvents without a co-solvent, such as propylene glycol methyl ether acetate (PGEMA), and a removing agent (dimethyl amino)trimethylsilane (DMTS) in the lift-off process. The photolithographic pixel patterning enables the exclusive positioning of EML materials at the desired places which are defined by the selective removal of the PR film through ultraviolet (UV) light exposure and fluorous solvent wash. Through this approach, the photo-patterning of 15 μm light-emitting pixels were achieved. A step further, red and green two-color pixels on a single substrate could finally be demonstrated with the acquisition of a photoluminescence (PL) image and an electroluminescence (EL) image of the configured pixels. This result strongly implies that OLED pixel-patterning using photolithography can be a promising candidate applicable to the production of high-resolution display panels.

## 2. Materials and Methods

### 2.1. Materials and Equipment

Negative-tone fluorinated photoresist, RF’-calix-O^t^Boc was synthesized according to literature [28]. CGI-1907 (photoacid generator, PAG) was purchased from BASF (Ludwigshafen, Germany). DMTS (>95%), and tris(trimethylsilyl)silane (>90%, TTMSS) were obtained from TCI (Tokyo, Japan) and used without purification. 1,1,1,2,3,4,5,6,6,6-Decafluoro-3-methoxy-4-trifluoromethylpentane (HFE-7300) was purchased from Kemis (Hwaseong, Korea) which is a distributor of 3M (St. Paul, MN, USA) in Korea. 1,4,5,8,9,11-Hexaazatriphenylene hexacarbonitrile (HATCN), N,N′-di(1-naphthyl)-N,N′-diphenyl-(1,1′-biphenyl)-4,4′-diamine (NPB), tris[2-phenylpyridine-C2,N]iridium(III) [Ir(ppy)_3_] were purchased from OSM (Goyang, Korea). Tris(4-carbazoyl-9-ylphenyl)amine (TCTA) was obtained from TCI (Tokyo, Japan) and used with purification. 1,3,5-tri(m-pyridin-3-ylphenyl)benzene (TmPyPB) was purchased from Aldrich (St. Louis, MO, USA) and tris(1-phenylisoquinoline)iridium(III) [Ir(piq)_3_] from Lumtec (New Taipei, Taiwan). Lithium fluoride (LiF) and aluminum (Al) was purchased from iTASCO (Seoul, Korea).

Surface treatment was carried out using plasma equipment (CUTE-1MPR manufactured by Femto Science Inc., Hwaseong, Korea). A 352 nm ultraviolet light-emitting diode (UV-LED, LABSYS LIT-2000 lithography system manufactured by NEXTRON, Busan, Korea) was used for UV irradiation. Spin coater (POLOS Spin 150i, APT Automation, Bünde, Germany) in a glove box was used for PR coating.

The current-voltage-luminance characteristics are measured using a source meter (B2912A, Agilent technology Inc., Santa Clara, CA, USA) and a National Institute of Standards and Technology (NIST) calibrated Si-photodiode FDS1010 (THORLABS Inc., Newton, NJ, USA) following standard procedures reported by Forrest et al. [29]. Electroluminescence spectra are obtained by a spectroradiometer (CS-2000, Minolta, Tokyo, Japan). An optical micrograph is taken using an optical microscope (Axio scope. A1, Carl Zeiss, Oberkochen, Germany).

### 2.2. Photolithographic Pixel Patterning

**Figure 1:**
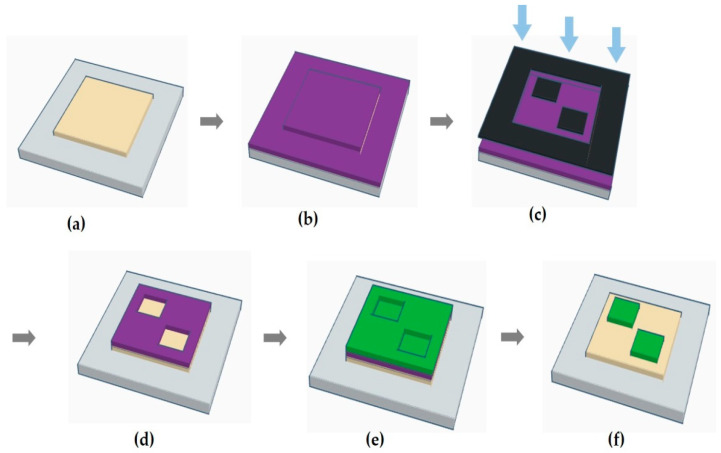
Schematic illustration of the photolithographic patterning process: (**a**) hole-transport layer (HTL) deposition on the indium tin oxide (ITO) substrate; (**b**) spin-coating of the photoresist (PR); (**c**) ultraviolet (UV) exposure through a photomask; (**d**) development of the photoresist film; (**e**) light-emitting layer (EML) deposition; (**f**) lift-off of the resist film and organic layer on it.

Figure 1 shows the steps involved in the lithographic patterning of two-color light-emitting pixels. In step (a), a HTL is thermally evaporated on a patterned indium tin oxide (ITO) substrate in a vacuum chamber (10^−7^ to 10^−8^ Torr). This is followed by the spin-coating of a highly fluorinated PR solution onto the HTL substrate under an N_2_ atmosphere in step (b). Spin coating was carried out at 1500 rpm for 1 min. The PR solution [28] used in the present work consists of a negative-tone material (RF’-calix-O^t^Boc) and PAG (CGI-1907) in HFE-7600 (3M) and the PR layer thickness is about 120–150 nm. After spin-coating, the substrate is soft-baked at 60 °C for 1 min under an N_2_ atmosphere. In step (c), the PR pattern is formed by UV exposure in air using a chrome mask with the desired pixel patterns. The irradiated substrate is then post-exposure baked (PEB) at 65 °C for 1 min in the air and the development process is performed in the air [step (d)]. During the development, the substrate is immersed in a fluorous solvent (HFE-7300, 3M), for 2 min to which a trace amount [0.005%(*v*/*v*)] of DMTS is added prior to use. Another rinsing step is carried out by dipping the substrate in HFE-7300 again for 1 min to which a trace amount [0.1%(*v*/*v*)] of TTMSS is added [30]. After development, a PR pattern is obtained for two-pixel formation along the diagonal line. An EML of a desired color is then deposited in the vacuum chamber [step (e)]. In some cases, an electron-transport layer (ETL) is deposited at the same time for the protection of the EML. Finally, in step (f), a lift-off step to remove the PR layer and organic film placed on it is carried out in the air by immersing the substrate in a fluorous solution for 3 min. HFE-7300 with an addition of an appropriate amount [1–3%(*v*/*v*) as the various thickness of stacked OLED layers] of DMTS is employed for this treatment. As a result, the substrate obtains a patterned EML (3.5 × 3.5 mm^2^) while the HTL is exposed in other parts of the substrate. Because of the concern about a possible increase in the driving voltage due to the PR residue, the substrate is rinsed with fresh HFE-7300 for 3 min. Then, the PR coating, soft-baking at the same condition was carried out to build the second color emitting pixels. UV exposure was performed using a chrome mask with the opposite pattern for the first emitting pixel. The development using same fluorous solvents, second EML deposition, and lift-off process are repeated. After the photo-patterning process, the electron injection layer (EIL) and metal cathode are deposited on the patterned substrate.

### 2.3. Organic Light-Emitting Diode (OLED) Fabrication

**Figure 2:**
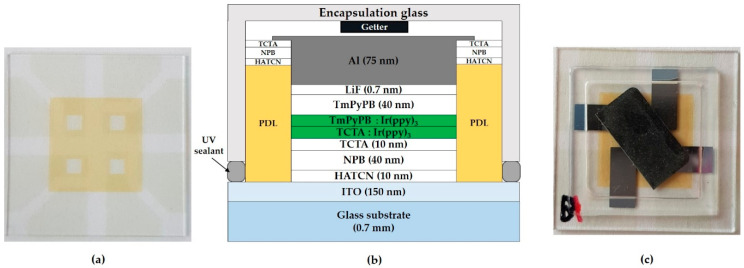
(**a**) ITO glass substrate image with patterned ITO and pixel-defined layer (PDL); (**b**) structure of green pixel patterned electroluminescent device used in this work; (**c**) the image of the encapsulated organic light-emitting diode (OLED) substrate covered with a moisture remover-containing glass lid.

ITO glass substrates (AMG, Korea) used in this work (Figure 2a) are patterned with 150 nm-thick of ITO and 1 μm-thick of polyimide (PI) bank acting as the pixel-defined layer (PDL). They are cleaned by ultrasonication in distilled water for 10 min, and dried in the air. Prior to organic layer deposition, O_2_ plasma surface treatment is performed for 5 min. The organic and metal layer deposition is then carried out by thermal evaporation in an ultra-high vacuum (10^−7^ to 10^−8^ Torr). The evaporation rates and layer thickness are measured by quartz crystal microbalance. The device consists of the following consecutive stacks: ITO, hole-injection layer (HIL), HTL, EML, ETL, EIL, and metal cathode. In detail, the 10 nm-thick HIL is composed of HATCN; the 50 nm-thick HTL consists of 40 nm film of NPB and 10 nm film of TCTA; the green EML is composed of TCTA and TmPyPB as the host material, which is doped with Ir(ppy)_3_. The thickness and doping concentration of the EML layer vary according to the experimental batch. The 40 nm-thick ETL is composed of TmPyPB, and the 0.7 nm-thick EIL consists of lithium fluoride (LiF). The 75 nm-thick cathode is composed of aluminum (Al). All the completed devices are encapsulated in an N_2_ atmosphere for measurement outside the glovebox (Figure 2b). Encapsulating glass lids possessing a moisture remover (HD type made of CaO, DYNIC, Tokyo, Japan) was placed over the OLED substrate and fixed with UV sealant resin (Figure 2c).

## 3. Results and Discussion

### 3.1. Development Process on Hole-Transport Layer (HTL)

To verify the applicability of the proposed photo-patterning scheme to the OLED pixel construction, we first examined the effect of the lithographic conditions upon the HTL because the PR pattern formation was carried out on top of it. After coating a PR layer on the deposited HTL substrate, the whole surface excluding the two diagonally placed rectangles was irradiated by UV light. The substrate was washed in a fluorous solution containing chemical additives (0.005%(*v*/*v*) of DMTS in HFE-7300 and 0.1%(*v*/*v*) of TTMSS in HFE-7300) the formulation of which had been reported in our previous study [30].

**Figure 3:**
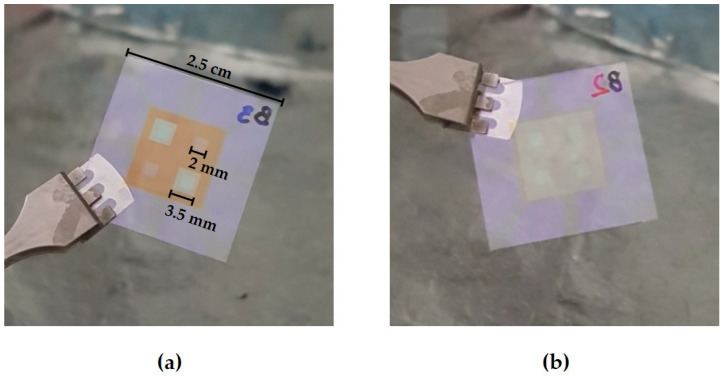
Photographic images of (**a**) the patterned device substrate after photoresist (PR) development and (**b**) the reference device substrate.

Figure 3a shows the processed substrate in which the two non-irradiated pixels along the left diagonal are not covered by the PR layer and thus have the color of the HTL. These two pixels constituted the light-emitting regions. The device fabrication was then completed by successive thermal evaporation of the EML, ETL, EIL, and cathode without the lift-off process of the PR layer. The EML stacks used in this batch consisted of TCTA:Ir(ppy)_3_ (15 nm, dopant concentration 8%) and TmPyPB:Ir(ppy)_3_ (15 nm, dopant concentration 8%). To evaluate the device characteristics, a reference device was also fabricated, which did not undergo the patterning work but was exposed to the air after the HTL deposition. Figure 3b shows the reference one in which the four pixels defined by the PDL have the color of the HTL.

**Figure 4:**
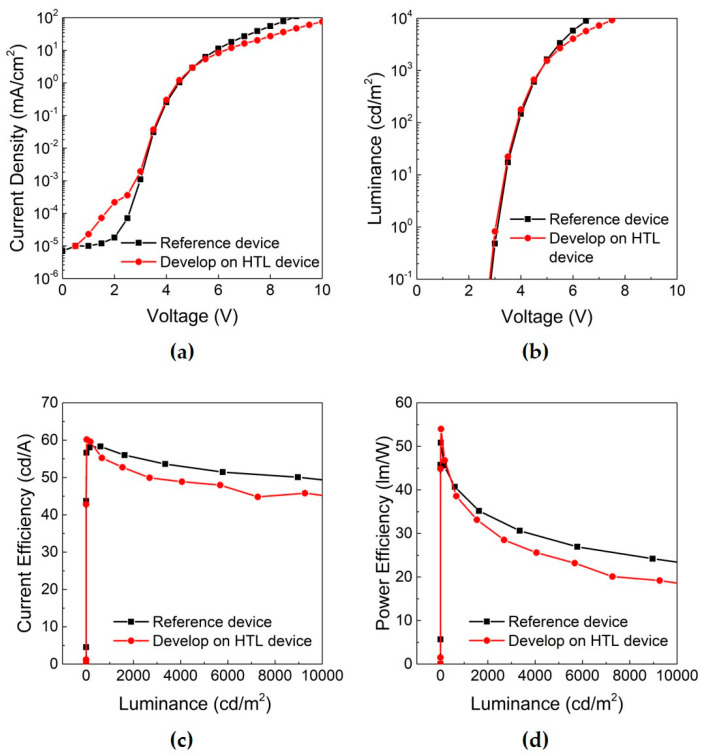
Comparison of the current-voltage-luminance (JVL) characteristics of the OLED patterned on the HTL (red line) and the reference (black line): (**a**) current density-voltage; (**b**) luminance-voltage; (**c**) current efficiency-luminance; (**d**) power efficiency-luminance.

Typical current-voltage-luminance (JVL) characteristics of the patterned and reference devices are presented in Figure 4a–d. As summarized in Table 1, the current efficiency of the patterned device was 54.3 cd/A at 1000 nit (cd/m^2^), which is around 95% of that of the reference device. Data of the other devices in a same batch are shown in a Supplementary Materials (Figure S1 and Table S1). Furthermore, the power efficiency was 36.5 lm/W and the external quantum efficiency (EQE) was 15.6%. It could be concluded that no significant negative effect occurred during the PR coating, UV exposure, post-exposure bake and develop process using fluorous solvents.

### 3.2. Lift-Off Process

After formation of a PR pattern on the HTL and deposition of the 10 nm-thick EML and the 40 nm-thick ETL, we attempted the lift-off step to remove the PR layer, EML, and ETL placed on top of it. Here, the EML stack consisted of TCTA:Ir(ppy)_3_ (5 nm, dopant concentration 8%) and TmPyPB:Ir(ppy)_3_ (5 nm, dopant concentration 8%). By immersing it into HFE-7300 containing an appropriate amount of DMTS [2.5%(*v*/*v*)], the lift-off process could be completed within 6 min in the air. To minimize the PR residue in the pixel area, the substrate is rinsed with fresh HFE-7300 and washed again with a syringe. In case of a previous bi-layer resist approach, around 2 h treatment in HFE solvent was required for lift-off [21]. For performance comparison, a reference substrate was also prepared by leaving it in the air without the patterning steps.

**Figure 5:**
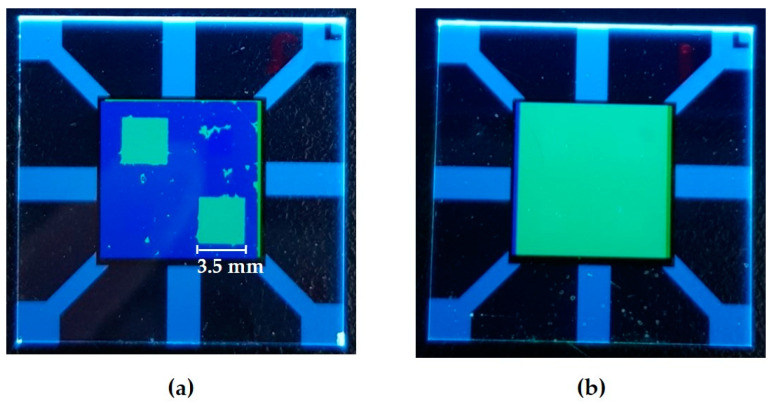
Photographic images of (**a**) the processed device after lift-off and (**b**) the reference device under an ultraviolet (UV) lamp.

**Figure 6:**
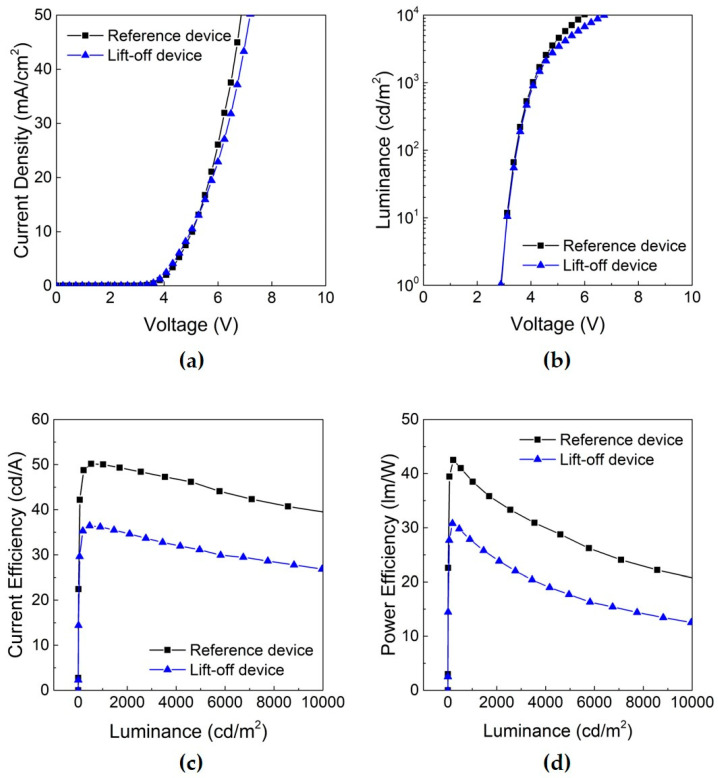
Current-voltage-luminance (JVL) characteristics of the OLED subjected to the lift-off process (blue line) and the non-processed reference (black line): (**a**) current density-voltage; (**b**) luminance-voltage; (**c**) current efficiency-luminance; (**d**) power efficiency-luminance.

The photographic image of the processed substrate, (a) in Figure 5, shows the patterned EML at the pixels along the left diagonal after the lift-off step under a UV lamp. In the case of the reference one, the substrate was fully covered by the EML, (b) in Figure 5. Examination of the JVL characteristics in Figure 6 indicates that the current efficiency of the processed device was 36.1 cd/A, which is at the 72% level of the reference device. The power efficiency of the processed device was 27.5 lm/W, and the EQE was 10.5% at 1000 nit. The performance of each device is summarized in Table 2. The operating voltage of the processed device was comparable to that of the reference one. Thus, it could be confirmed that photo-patterning of single-color pixels on top of the HTL can reach as high as 72% performance level of the non-patterned reference without the operating voltage deteriorating much. Other devices that have gone through lift-off steps under slightly different conditions, including different exposure time and stripper concentration, show nearly similar performances to the lift-off processed device using 2.5% of DMTS (Figure S2 and Table S2). It was assumed that the performance degradation after the lift-off step could be associated with the several percent concentration of the removing agent DMTS. Furthermore, it was found that an effective rinsing with fresh solvent was necessary to minimize the increase in operating voltage that could occur due to the deposition of the PR residue.

**Figure 7:**
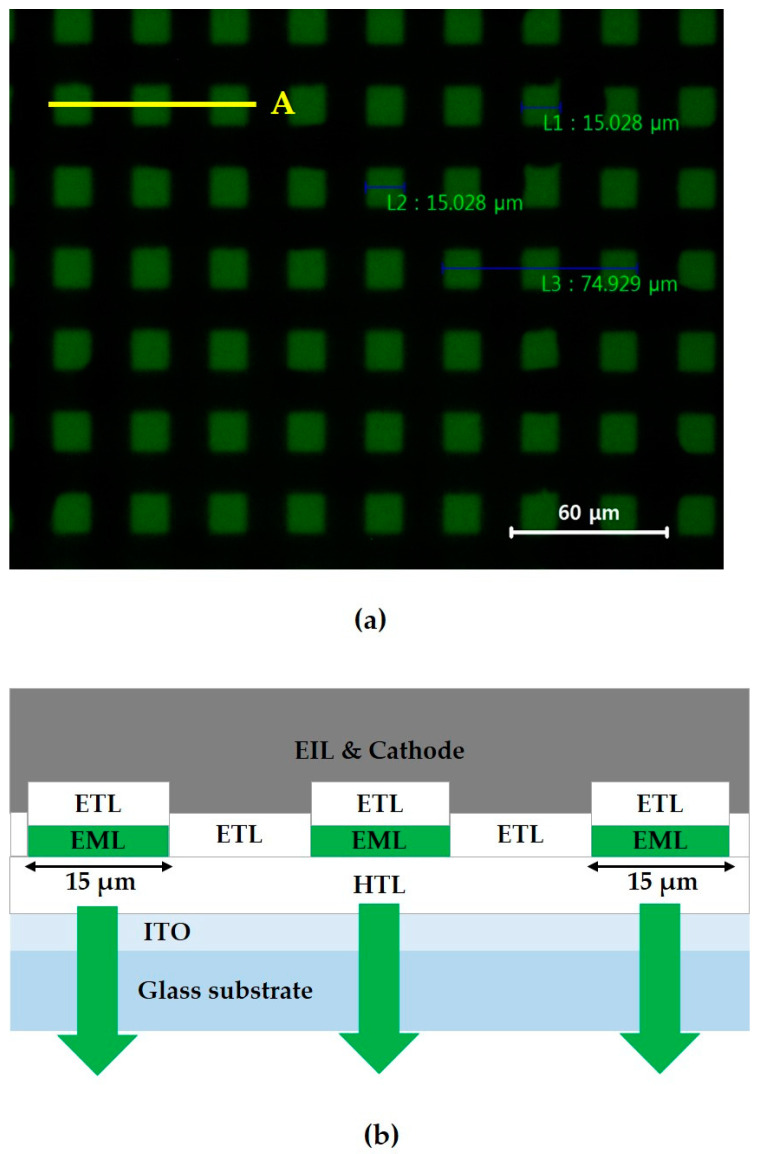
(**a**) Optical micrograph of the 15 μm green-emitting pixel array during the OLED operation; (**b**) stack structure of the OLED indicated by the segmental line A in picture (**a**).

The photo-patterning scheme was then examined in the construction of precisely defined emitting pixels of micrometer scale size. The optical micrograph in Figure 7a shows an EL image of the OLED device with the 15 μm photo-patterned EML, and Figure 7b depicts the OLED stack structure in the region indicated by the segmental line A in Figure 7a. In this case, the emitting area was not defined by the PDL pattern on the ITO, but by the photo-patterned EML itself on the common HTL. Inside a 2 × 2 mm^2^ region defined by the PDL, there is an array of 15 μm rectangles of the patterned EML with 30 μm pixel pitch. It is noteworthy that the lift-off step in the micrometer-scale pixel size regime could be carried out successfully, compared to the current state of the art in the FMM method of OLED pixel patterning [31,32].

### 3.3. Two-Color Pixel Patterning

**Figure 8:**
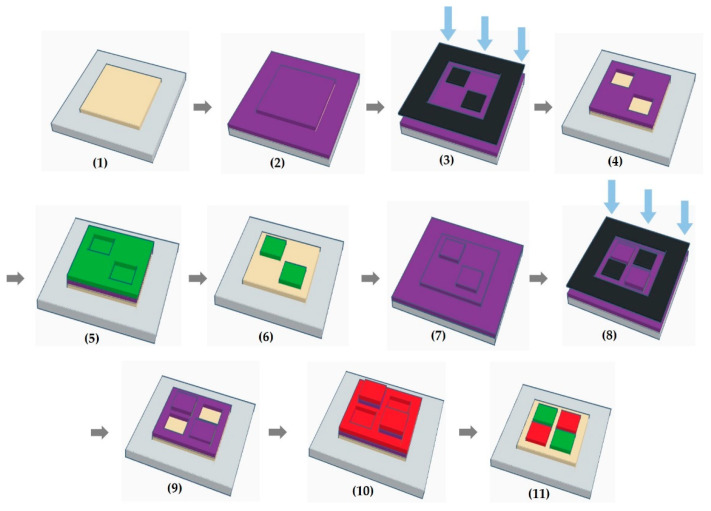
Schematic illustration of the photolithographic patterning process about two-color pixel patterning.

**Figure 9:**
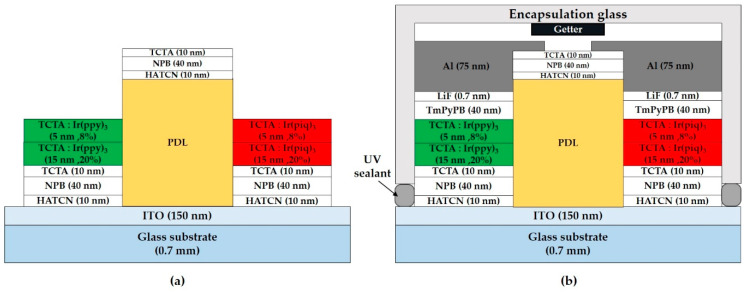
(**a**) Stack structure of green and red patterned EML after second lift-off; (**b**) structure of two-color pixel patterned electroluminescent device.

Two-color light-emitting pixel patterning was undertaken on the same substrate by repeating the single-color patterning process as shown in Figure 8 in order to verify the potential of our approach. We encountered a difficult situation for a second PR coating on first patterned green EML and ETL. The process step is shown from (6) to (7) in Figure 8. Due to the difference in wettability of the PR solution on HTL and ETL materials, a PR film could not be perfectly coated on the first patterned EML/ETL. As shown in Figure 9a, we used a single TCTA host instead of double TCTA/TmPyPB hosts into a green EML to resolve the de-wetting problem of the PR solution in the second photolithography process. Therefore, the lift-off step was performed on top of the EML rather than on EML/ETL. After lift-off of the red EML, substrate was patterned with two-color EMLs as step (11) of Figure 8. Then, the ETL, EIL, and metal cathode were deposited and encapsulated in an N_2_ atmosphere with a moisture remover and UV sealant resin (Figure 9b). To achieve preliminary working two-color light-emitting pixels, the EML composition and structure was slightly modified with an adjusted stripper concentration.

**Figure 10:**
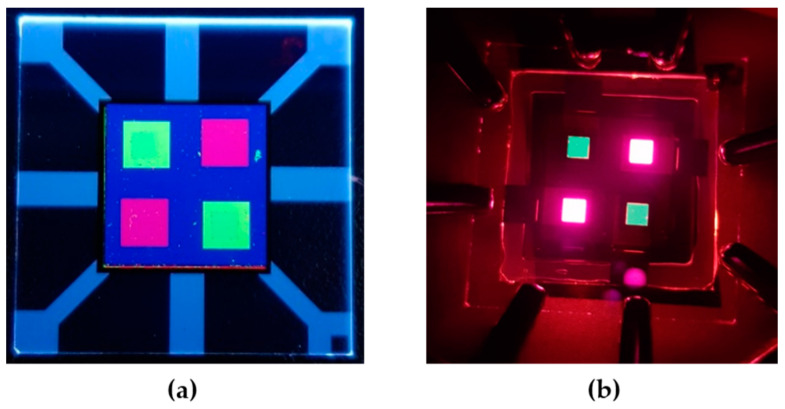
(**a**) Photoluminescence (PL) image of the processed device under UV lamp excitation; (**b**) electroluminescence (EL) image of the device after two-color pixel patterning.

The PL image in Figure 10a shows the diagonally patterned, two different EMLs (3.5 × 3.5 mm^2^). Figure 10b presents the actual operating pixels of the device with the photo-patterned two-color EMLs. The emitting area (2 × 2 mm^2^) is smaller than that of the patterned EMLs (3.5 × 3.5 mm^2^) because it was further defined by the PDL inside the EML patterns on top of the ITO electrode. Further study is aimed at finding conditions to increase the efficiency of the first patterned light-emitting pixels which undergo two lift-off steps. This includes the implementation of a new process scheme and materials, such as applying a protective layer on top of the patterned pixel and performing a lift-off step with a benign additive.

## 4. Conclusions

In this study, we demonstrated the two-color OLED pixels integrated on a single substrate using a negative-tone highly fluorinated PR and fluorous solvents performed on a common HTL. Preliminary experiments were performed to examine the probable damaging effects of the developing and lift-off steps on a HTL. No significant deterioration in the efficiency of the develop-processed device was observed at 1000 nit. The processed device after lift-off achieved an efficiency of up to 72% relative to the reference device at 1000 nit, with no change in the operating voltage. The same procedure for the single color patterning was repeated once more to successfully obtain two-color pixel arrays. In addition, the patterning of 15 μm green pixels was accomplished. These results tell us that photolithography can provide a useful tool for the production of high-resolution large OLED displays in the near future.

## Figures and Tables

**Table 1:** Performance summary of a reference device and a develop processed device.

Processing Stage	Device	Current Efficiency ^1^ (cd/A)	Power Efficiency ^1^ (lm/W)	External Quantum Efficiency ^1^ (%)	Driving Voltage ^1^ (V)	Turn on Voltage ^2^ (V)
Development on HTL	Reference	57.4	38.6	16.5	4.7	3.0
	Processed	54.3	36.5	15.6	4.7	3.0

^1^ at 1000 nit, ^2^ at 1 nit.

**Table 2:** Performance summary of a reference device and a processed device after lift-off.

Processing Stage	Device	Current Efficiency ^1^ (cd/A)	Power Efficiency ^1^ (lm/W)	External Quantum Efficiency ^1^ (%)	Driving Voltage ^1^ (V)	Turn on Voltage ^2^ (V)
Lift-off	Reference	50.0	38.6	14.5	4.0	2.8
	Processed	36.1	27.5	10.5	4.1	2.8

^1^ at 1000 nit, ^2^ at 1 nit.

## Supplementary Materials

The following are available online at https://www.mdpi.com/2072-666X/11/7/650/s1: Figure S1: Additional comparison of the current-voltage-luminance (JVL) characteristics of the OLED patterned on the HTL devices and the reference devices: (a) current density-voltage; (b) luminance-voltage; (c) current efficiency-luminance; (d) power efficiency-luminance, Table S1: Additional performance summary of reference devices and develop processed devices, Figure S2: Additional comparison of the current-voltage-luminance (JVL) characteristics of the OLED subjected to the lift-off process devices and the non-processed reference devices: (a) current density-voltage; (b) luminance-voltage; (c) current efficiency-luminance; (d) power efficiency-luminance, Table S2: Additional performance summary of reference devices and lift-off devices (slight different conditions for exposure time and stripper concentration).

## References

[1] Tang C.W., VanSlyke S.A. (1987). Organic electroluminescent diodes. Appl. Phys. Lett..

[2] Baldo M.A., O’Brien D.F., You Y., Shoustikov A., Sibley S., Thompson M.E., Forrest S.R. (1998). Highly efficient phosphorescent emission from organic electroluminescent devices. Nature.

[3] Adachi C., Baldo M.A., Thompson M.E., Forrest S.R. (2001). Nearly 100% internal phosphorescence efficiency in an organic light-emitting device. Appl. Phys. Lett..

[4] Reineke S., Lindner F., Schwartz G., Seidler N., Walzer K., Lüssem B., Leo K. (2009). White organic light-emitting diodes with fluorescent tube efficiency. Nature.

[5] Kim J.W., Choe W.J., Hwang K.H., Kwag J.O. (2017). The Optimum Display for Virtual Reality. SID Symp. Dig. Tech. Pap..

[6] Sugimoto A., Ochi H., Fujimura S., Yoshida A., Miyadera T., Tsuchida M. (2004). Flexible OLED displays using plastic substrates. IEEE J. Sel. Top. Quantum Electron..

[7] Yin D., Feng J., Ma R., Liu Y.F., Zhang Y.L., Zhang X.L., Bi Y.G., Chen Q.D., Sun H.B. (2016). Efficient and mechanically robust stretchable organic light-emitting devices by a laser-programmable buckling process. Nat. Commun..

[8] Bardsley J.N. (2004). International OLED technology roadmap. IEEE J. Sel. Top. Quantum Electron..

[9] Sasaki T., Yamaoka R., Nomura S., Yamamoto R., Takahashi K., Nakamura D., Aoyama T., Ikeda H., Seo S., Yamazaki S. (2017). A 13.3-inch 8K x 4K 664-ppi 120-Hz 12-bit Display with Super-wide Color Gamut for the BT.2020 Standard. SID Symp. Dig. Tech. Pap..

[10] Shiokawa M., Toyotaka K., Tsubuku M., Sugimoto K., Nakashima M., Matsuda S., Shishido H., Aoyama T., Ikeda H., Eguchi S.A. (2016). 1058 ppi 8K4K OLED Display using a Top-Gate Self-Aligned CAAC Oxide Semiconductor FET. SID Symp. Dig. Tech. Pap..

[11] Lih J.J., Chao C.I., Lee C.C. (2007). Novel pixel design for high-resolution AMOLED displays with a shadow mask. J. SID.

[12] Kwon J.H. (2013). RGB color patterning for AMOLED TVs. Inf. Disp..

[13] Laaperi A. (2008). OLED lifetime issues from a mobile-phone-industry point of view. J. SID.

[14] Chang S.C., Liu J., Bharathan J., Yang Y., Onohara J., Kido J. (1999). Multicolor Organic Light-Emitting Diodes Processed by Hybrid Inkjet Printing. Adv. Mater..

[15] Kang Y.J., Bail R., Lee C.W., Chin B.D. (2019). Inkjet Printing of Mixed-Host Emitting Layer for Electrophosphorescent Organic Light-Emitting Diodes. ACS Appl. Mater. Interfaces.

[16] Shtein M., Peumans P., Benziger J.B., Forrest S.R. (2004). Direct, Mask- and Solvent-Free Printing of Molecular Organic Semiconductors. Adv. Mater..

[17] Quinn W.E., McGraw G.J., Harikrishna-Mohan S., Kottas G.S., King M., Swedlove B., Kantor J., Trojacki T., Brown J.J. (2017). Organic Vapor Jet Printing, a Solvent-Less, Mask-Less Patterning Technology for OLED Displays. SID Symp. Dig. Tech. Pap..

[18] Wolk M.B., Baetzold J.P., Bellmann E., Hoffend T.R., Lamansky S., Li Y., Roberts R.R., Savvateev V., Staral J.S., Tolbert W.A. Laser thermal patterning of OLED materials. Proceedings of the Organic Light-Emitting Materials and Devices VIII.

[19] Cha S.J., Jeon J.H., Suh M.C. (2014). Full color organic light emitting dioldes with laser-patterned optical path-length compensation layer. Org. Electron..

[20] Hirano T., Matsuo K., Kohinata K., Hanawa K., Matsumi T., Matsuda E., Matsuura R., Ishibashi T., Yoshida A., Sasaoka T. (2007). Novel Laser Transfer Technology for Manufacturing Large-Sized OLED Displays. SID Symp. Dig. Tech. Pap..

[21] Krotkus S., Ventsch F., Kasemann D., Zakhidov A.A., Hofmann S., Leo K., Gather M.C. (2014). Photo-patterning of Highly Efficient State-of-the-Art Phosphorescent OLEDs Using Orthogonal Hydrofluoroethers. Adv. Opt. Mater..

[22] Krotkus S., Nehm F., Janneck R., Kalkura S., Zakhidov A.A., Schober M., Hild O.R., Kasemann D., Hofmann S., Leo K. Influence of bilayer processing on p-i-n OLEDs: Towards multicolor photolithographic structuring of organic displays. Proceedings of the Organic Photonic Materials and Devices XVII.

[23] Malinowski P.E., Ke T., Nakamura A., Chang T.Y., Gokhale P., Steudel S., Janssen D., Kamochi Y., Koyama I., Iwai Y. (2015). True-Color 640 ppi OLED Arrays Patterned by CA i-line Photolithography. SID Symp. Dig. Tech. Pap..

[24] Gather M.C., Köhnen A., Falcou A., Becker H., Meerholz K. (2007). Solution-Processed Full-Color Polymer Organic Light-Emitting Diode Displays Fabricated by Direct Photolithography. Adv. Funct. Mater..

[25] Zakhidov A.A., Lee J.K., Fong H.H., DeFranco J.A., Chatzichristidi M., Taylor P.G., Ober C.K., Malliaras G.G. (2008). Hydrofluoroethers as orthogonal solvents for the chemical processing of organic electronic materials. Adv. Mater..

[26] Taylor P.G., Lee J.K., Zakhidov A.A., Chatzichristidi M., Fong H.H., DeFranco J.A., Malliaras G.G., Ober C.K. (2009). Orthogonal Patterning of PEDOT:PSS for Organic Electronics using Hydrofluoroether Solvents. Adv. Mater..

[27] Lee J.K., Chatzichristidi M., Zakhidov A.A., Taylor P.G., DeFranco J.A., Hwang H.S., Fong H.H., Holmes A.B., Malliaras G.G., Ober C.K. (2008). Acid-Sensitive Semiperfluoroalkyl Resorcinarene: An Imaging Material for Organic Electronics. J. Am. Chem. Soc..

[28] Son J., Oh H.T., Kwon O.J., Lim J.M., Jung H., Jung B.J., Hwang D.H., Lee C., Lee J.K., Yoon J.G. (2017). Highly soluble fluorous alkyl ether-tagged imaging materials for the photo-patterning of organic light-emitting devices. J. Mater. Chem. C.

[29] Forrest S.R., Bradley D.D., Thompson M.E. (2003). Measuring the Efficiency of Organic Light-Emitting Devices. Adv. Mater..

[30] Son J., Shin H.Y., Choi Y.M., Chae S.G., Park C., Lee J.K., Jung B.J. (2020). Descumming fluorous solution for photolithographic patterning of organic light-emitting diodes. Microelectron. Eng..

[31] Toppan Printing Co., Ltd. Electronics Division. http://www.toppan.co.jp/electronics/english/display/oled_metal_mask.

[32] Heo J., Min H., Lee M. (2015). Laser Micromachining of Permalloy for Fine Metal Mask. Int. J. Precis. Eng. Manuf.-Green Technol..

