# Supplementary material for: Two-Color Pixel Patterning for High-Resolution Organic Light-Emitting Displays Using Photolithography
Source: Micromachines (Basel). 2020 Jun 30;11(7):650. doi: 10.3390/mi11070650 (PMC7408551; doi:10.3390/mi11070650)
Supplement: Supplementary file 1 [file micromachines-11-00650-s001.pdf]

micromachines

Supplementary Materials: Two-Color Pixel Patterning for High Resolution Organic Light-Emitting Displays Using Photolithography

Yu Min Choi [1], Han Young Shin [1], Jongchan Son [2], Chunhee Park [2], Keun-Woo Park [2], Jin-Kyun Lee [2,*] and Byung Jun Jung [1,*]

[1] Department of Materials Science and Engineering, University of Seoul, Seoul 02504, Republic of Korea; ccwe1001@naver.com (Y.M.C.); tlsgksdud24@naver.com (H.Y.S.)

[2] Department of Polymer Science and Engineering, Inha University, Incheon 22212, Republic of Korea; thswhdcks1@naver.com (J.S.); nhd8991@naver.com (C.P.); rmsdn0112@naver.com (K.-W.P.)

* Correspondence: jkl36@inha.ac.kr (J.-K.L.); jungbj@uos.ac.kr (B.J.J.); Tel.: +82-32-860-7481 (J.-K.L.); +82-2-6490-2412 (B.J.J.)

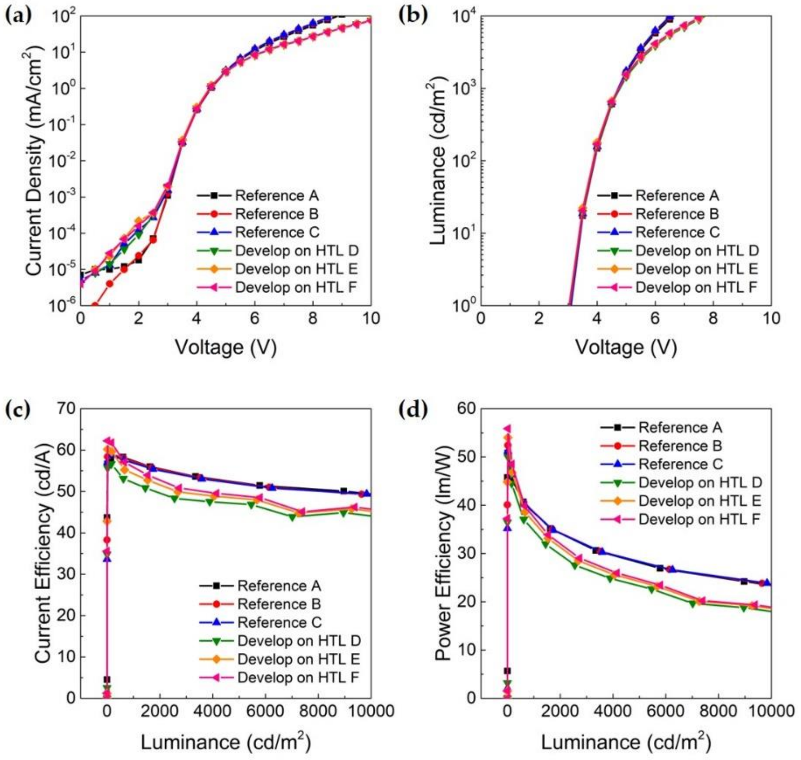

Figure S1. Additional comparison of the current-voltage-luminance (JVL) characteristics of the OLED patterned on the HTL devices and the reference devices: (**a**) current density-voltage; (**b**) luminance-voltage; (**c**) current efficiency-luminance; (**d**) power efficiency-luminance.

Table S1. Additional performance summary of reference devices and develop processed devices.

Device	Current efficiency [1] (cd/A)	Power efficiency [1] (lm/W)	External quantum efficiency [1] (%)	Driving voltage [1] (V)	Turn on voltage [2] (V)
Reference A	57.4	38.6	16.5	4.7	3.0
Reference B	57.3	39.0	16.4	4.7	3.0
Reference C	57.0	38.5	16.3	4.7	3.0
Develop on HTL D	52.1	34.7	15.0	4.7	3.0
Develop on HTL E	54.3	36.5	15.6	4.7	3.0
Develop on HTL F	55.9	37.6	16.0	4.7	3.0

[1] at 1000 nit, [2] at 1 nit.

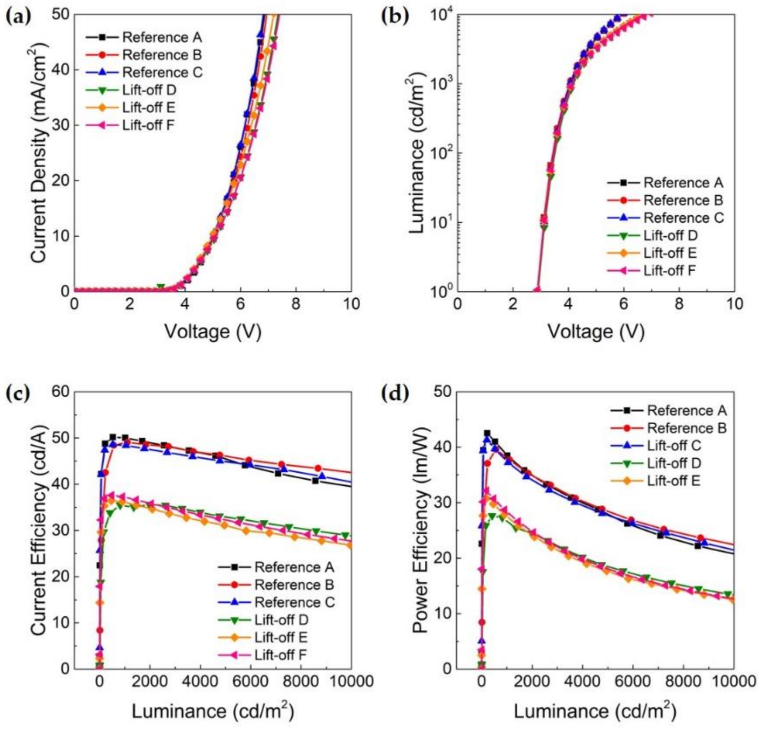

Figure S2. Additional comparison of the current-voltage-luminance (JVL) characteristics of the OLED subjected to the lift-off process devices and the non-processed reference devices: (**a**) current density-voltage; (**b**) luminance-voltage; (**c**) current efficiency-luminance; (**d**) power efficiency-luminance.

Table S2. Additional performance summary of reference devices and lift-off devices (slight different conditions for exposure time and stripper concentration).

Device (exposure time at 20 mW/cm^2, stripper concentration)	Current efficiency [1] (cd/A)	Power efficiency [1] (lm/W)	External quantum efficiency [1] (%)	Driving voltage [1] (V)	Turn on voltage [2] (V)
Reference A	50.0	38.6	14.5	4.0	2.8
Reference B	49.0	38.6	14.6	4.1	2.9
Reference C	48.4	37.5	14.1	4.1	2.9
Lift-off D (30s exposure, 3.0%(v/v))	35.3	26.7	10.3	4.2	2.9
Lift-off E (30s exposure, 2.5%(v/v))	36.1	27.5	10.5	4.1	2.8
Lift-off F (50s exposure, 3.0%(v/v))	37.2	28.4	11.0	4.1	2.9

[1] at 1000 nit, [2] at 1 nit.
